# Feasibility evaluation of N-Isopropyl Acrylamide 3D gel dosimeters for proton therapy

**DOI:** 10.1371/journal.pone.0291507

**Published:** 2023-09-12

**Authors:** Chun-Hsu Yao, Eng-Yen Huang, Kuo-Jung Juan, Pei-Jiuan Juang, Ying-Hsuan Juan, Yuan-Jen Chang

**Affiliations:** 1 Department of Master Program for Biomedical Engineering / School of Chinese Medicine, China Medical University, Taichung City, Taiwan, R.O.C; 2 Biomaterials Translational Research Center, China Medical University Hospital, Taichung City, Taiwan, R.O.C; 3 Department of Biomedical Imaging and Radiological Science, China Medical University, Taichung City, Taiwan, R.O.C; 4 School of Chinese Medicine, China Medical University, Taichung City, Taiwan, R.O.C; 5 Department of Biomedical Informatics, Asia University, Taichung City, Taiwan, R.O.C; 6 Department of Radiation Oncology, Chang Gung Memorial Hospital Kaohsiung Branch, Niao-Sung District, Kaohsiung, Taiwan, R.O.C; 7 Department of Aerospace and Systems Engineering, Feng-Chia University, Taichung, Taiwan, R.O.C; Brandeis University, UNITED STATES

## Abstract

This study aimed to investigate the feasibility of applying 3D gel dosimeters for proton therapy. Two different formulations (5-5-3-5, 5-3-3-10) for the N-Isopropyl Acrylamide (NIPAM) polymer gel were used to find the best composition for the application of NIPAM polymer gels for proton therapy. The reaction of the gel under different physical conditions, including dependence on energy and dependence on the dose rate of the NIPAM gel under proton irradiation, was also explored. A NIPAM gel dosimeter was used to record the 3D dose distribution, and a self-developed parallel beam optical computed tomography scanner was used to obtain non-irradiated and post-irradiated gel phantom images. The NIPAM gel was filled into a cylindrical acrylic phantom. The results showed that the optical density of the irradiated NIPAM dosimeter was linear in the dose range of 0 to 6 Gy, and the linearity of the two NIPAM gel formulations at the depth of the dose point (2 cm) was 0.98 to 0.89. The dose depth curves showed different patterns with different gel sensitivities. This study demonstrated that the NIPAM gel dosimeter with the 5-3-3-10 formulation is suitable for verifying the dosimetry dose of proton beams.

## Introduction

Radiation therapy is one of the methods for the clinical treatment of cancer. The main goal of radiation therapy is to improve tumor control rate and avoid normal tissue to radiation dose. Therefore, dose verification is an important part of radiation therapy.

With the advancement of radiation therapy in recent years, the number of patients who choose proton therapy increases [1, 2]. Proton therapy is an external radiation treatment that has special physical characteristics called Bragg Peak. When the beam passes through normal tissue, less energy is released until it reaches the depth of the tumor to be treated. The dose at the end of the proton beam is drastically decreased for normal tissue not to receive the residual dose.

Precise 3D dosimetry of proton beams is important to ensure tumor treatment with minimal damage to the healthy surrounding tissues. Only 1D or 2D dosimeters, such as an ionization chamber and a film dosimeter, are traditionally available in clinical radiation therapy. The polymer gel dosimeter is currently the only 3D dosimeter capable of measuring the 3D dose distribution [3]. It has high-dose resolution and accuracy. After the gel is irradiated by radiation, the monomer generates polymerization and cross-linking reaction, and it has high sensitivity and linearity. Therefore, this feature could be applied to the verification tools of clinical radiation doses. In the present study, the N-isopropylacrylamide gel dosimeter proposed by Senden et al. in 2006 was used [4]. NIPAM gel has low toxicity, high sensitivity, and high linearity, and it could be prepared under normoxic conditions.

Previous studies have confirmed that the NIPAM 3D gel dosimeter with OCT could provide good dose verification for photon therapy [5–8] and developed various combinations to obtain the sensitivity linearity formulas most suitable for photon therapy. Different gel formulations could be used to achieve the precision of NIPAM polymer gels for various clinical applications [9–11].

Many previous studies have also confirmed that, although the gel has excellent 3D scanning performance, the reaction of the gel dosimeter in the high Linear Energy Transfer (LET) [12–18] region of the dose distribution is reduced and the absorbed dose of the Bragg peak is not reproduced. Therefore, the dose measurement becomes complicated and makes the experiment more challenging.

The present study aimed to evaluate the feasibility of a 3D NIPAM polymer gel with optical computed tomography (CT) for proton therapy.

## Materials and methods

### Gel preparation

The gel dosimeter used in this study was the NIPAM polymer gel proposed by Senden et al. [4]. Previous studies have mentioned the sensitivity and linearity of different formulations [9–11]. The gel composition was gelatin (300 Bloom Type A, Sigma–Aldrich), NIPAM (97% pure; Wako, Osaka, Japan), N,N-methylene bisacrylamide (Bis) and tetrakis (hydroxymethyl) phosphonium chloride (THPC). The gel preparation process is as follows. In the beginning, the gel phantom was heated to 45°C in a water bath. Then 3 wt% Bis was added and dissolved at 45°C by continuous stirring for 15 min. Subsequently, 5 wt% monomer was added and dissolved by continuous stirring at 45°C for approximately 15 min. Finally, 5 mM THPC was added to the solution by continuous stirring for 2 min. The resulting gel was clear and transparent and poured into cylindrical acrylic phantoms. Two different formulations (5-5-3-5 and 5-3-3-10) of the NIPAM polymer gel were prepared and applied to proton therapy to identify the optimal composition of the NIPAM polymer gel, as shown in Table 1.

**Table 1:** Two different formulations of NIPAM polymer gel.

	Gelatin	NIPAM	Bis	THPC	Sensitivity
**5-5-3-5**	5%	5%	3%	5 mM	High
	(5 g)	(5 g)	(3 g)		
**5-3-3-10**	5%	3%	3%	10 mM	Low
	(5 g)	(3 g)	(3 g)		

The gel preparation process was as follows: first, gelatin was added to deionized water and heated to 45° C. Then Bis and NIPAM were added to the clear and transparent gelatin solution and stirring was continued for 30 minutes. Finally, THPC was added after the temperature was cooled to 42°C. After the preparation was complete, the solution was poured into a 10 × 10 acrylic cylindrical container and cooled in a 6° C water cooling system for at least 6 hours. The NIPAM gel was covered with aluminum foil paper to avoid photopolymerization.

### Irradiation

The treatment planning system used in this study was RayStation, RaySearch, version 6.2.0.7, provided by Kaohsiung Chang Gung Memorial Hospital.

After the gel was prepared, an energy of 100 MeV was fixed and the gel dosimeters of the two formulations were irradiated with a dose of 0 to 6 Gy to obtain a dose–response curve.

**Fig 1:**
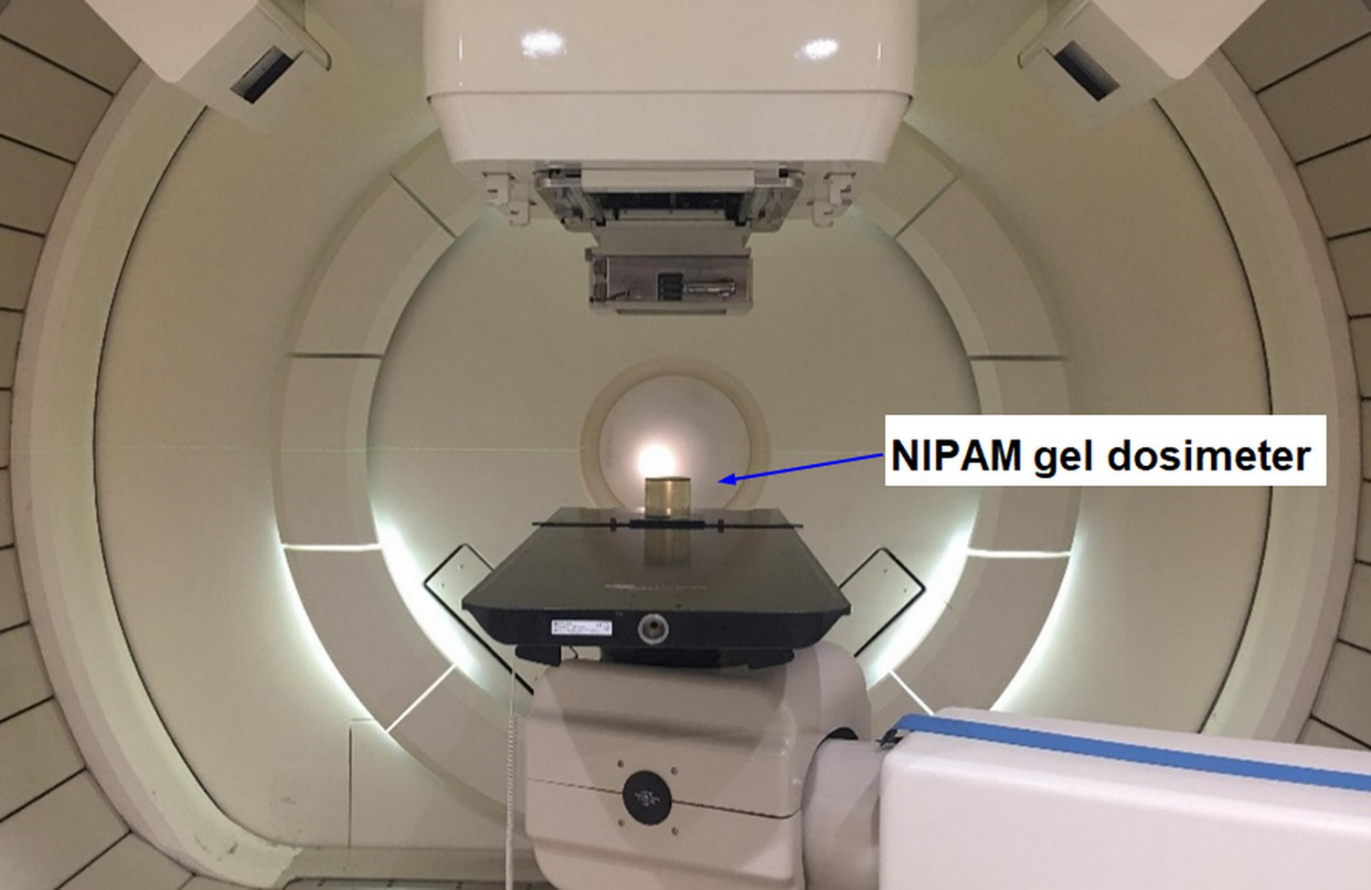
Proton beam irradiation system and NIPAM gel phantom.

Three different target doses (2, 4, and 6 Gy) were set to explore the dependence of the dose rate. For each dose value, three dose rates were set, including 20, 40, and 80 MU/S. Furthermore, three energies of proton beam (80, 100, and 120 MeV) were set to explore the energy dependence of the gel dosimeter and three different target doses with 0–3 Gy at each energy value. The irradiation parameters were shown in Table 2. All batches of NIPAM gel irradiation gantry angle were 0°, as shown in Fig 1.

**Table 2:** The irradiation parameters of a pencil beam line scanning.

Experiment	Target dose (Gy)	Dose rate (MU/s)	Energy (MeV)	gantry angle (°)
**Dose rate dependence**	0, 2, 4, 6	20, 40, 80	80, 100, 120	0
**Energy dependence**	0, 1, 2, 3	20, 40, 80	80, 100, 120	0

### Dose read-out tool

**Fig 2:**
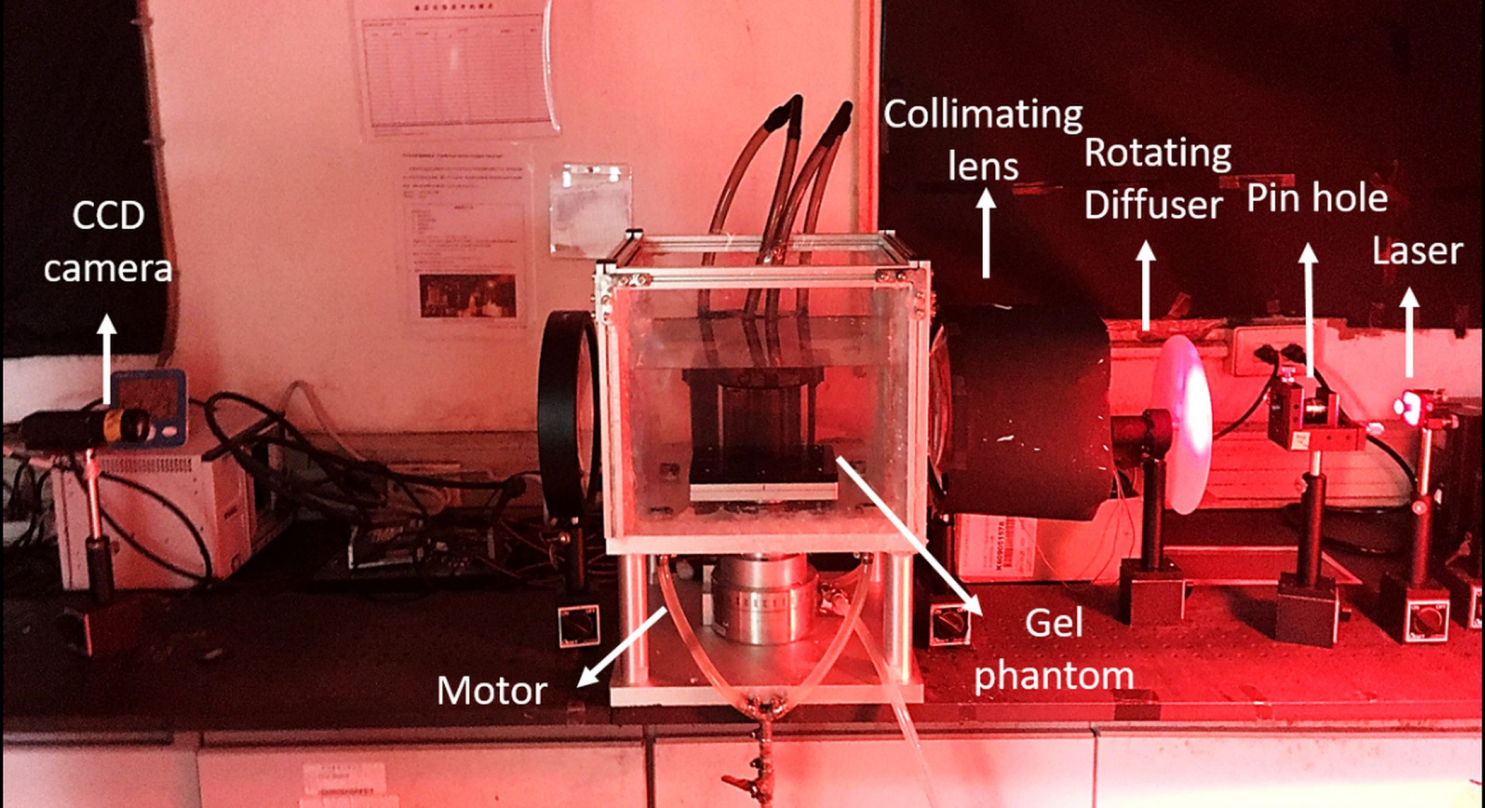
Optical CT scanner.

In this study, a parallel beam optical CT scanner based on a charge-coupled device (CCD) (CT-s2) [19] was used as the dose readout tool, as shown in Fig 2. It was used to acquire nonirradiated and post-irradiated gel phantom images.

During the scanning process, the gel was placed in the water tank filled with the matching liquid to prevent the laser light from refracting or scattering due to the different refractive indices of the medium.

The final step was to reconstruct the image and perform dose analysis using filtered back projection in MATLAB.

## Results and discussion

### Gel spatial uniformity

**Fig 3:**
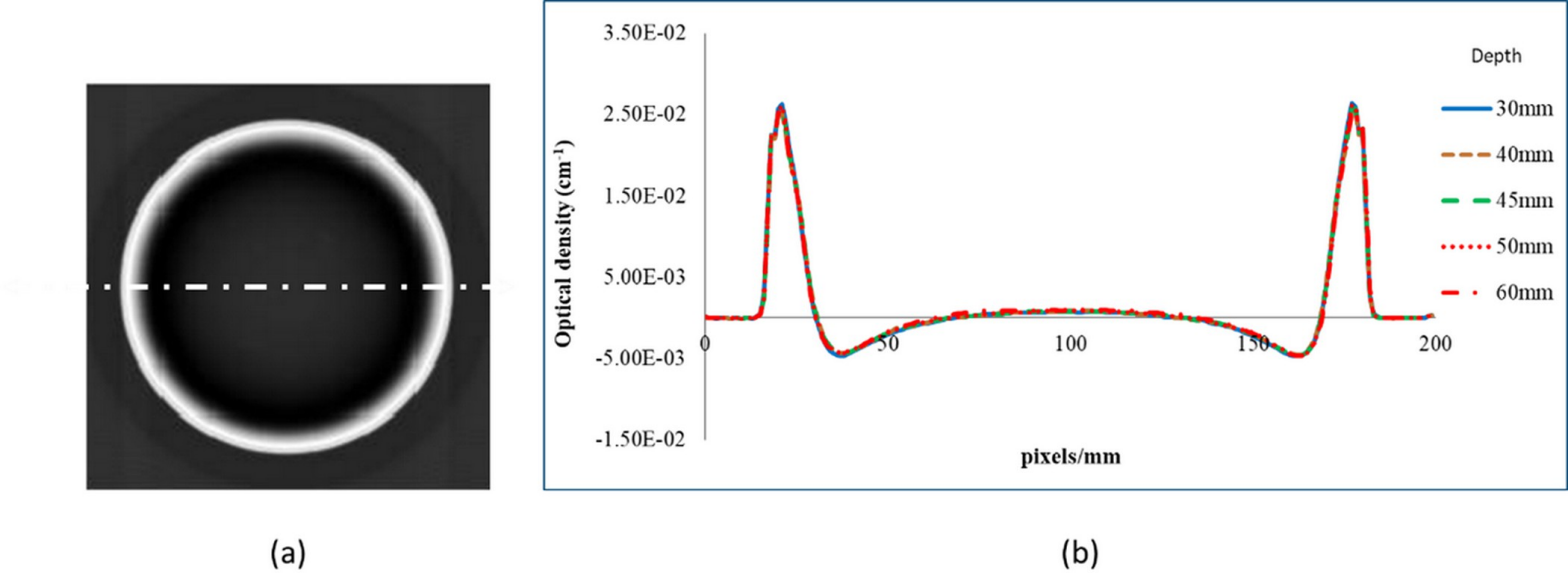
(a) Reference image of the NIPAM gel phantom. (b) Line profiles of the NIPAM gel phantom at depths of 30, 40, 45, 50, and 60 mm.

Before the NIPAM gel was irradiated, optical CT was used to obtain the pre-irradiation image. Fig 3 shows the scanning results of the preirradiation image. On the left side of the Fig 3 is a reconstructed image of the transverse of the gel. The right side of the Fig 3 shows the optical density (OD) at different depths of 30, 40, 45, 50, and 60 mm. Before irradiation, the gel center OD value of the gel was 0, and both sides were the walls of the gel phantom. Slices at different depths of layers 30, 40, 45, 50, and 60 mm are used to calculate gel uniformity. The uncertainties are defined as the ratio of the standard deviation to the mean value of OD. The uncertainty in the center 100 pixel/mm region covering from 50 to 150 pixel/mm was calculated. The average uncertainty of the total of 12 batches was less than 3% and the minimum uncertainty was 2%. This result confirmed that the uniformity of the gel phantom in this study was consistent.

### Dose–response curve

**Fig 4:**
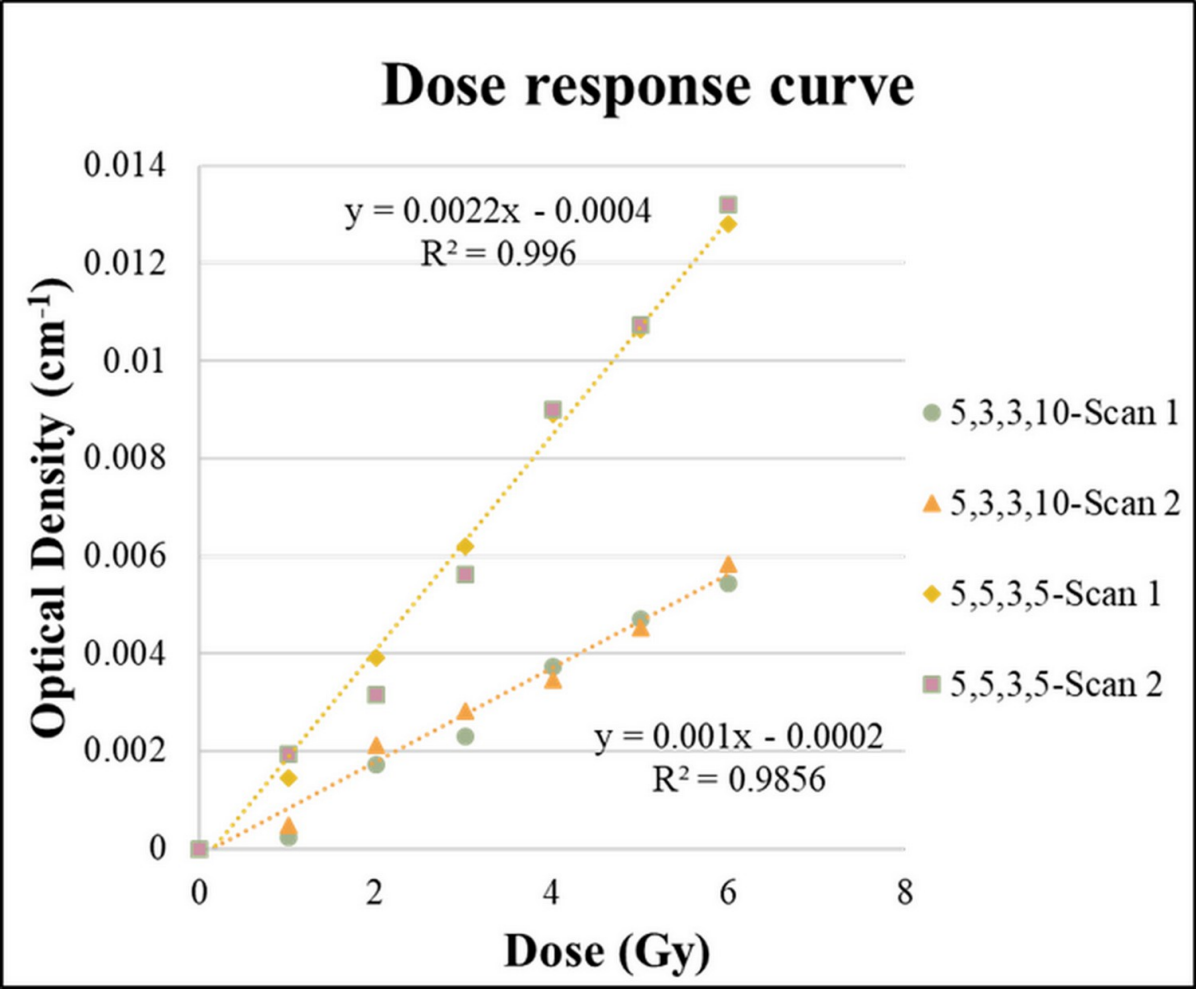
Dose–response curves of two different NIPAM gel formulations.

The relationship between the OD and the dose is called the dose–response curve. Fig 4 shows the dose–response curve of the NIPAM dosimeter with two formulations (5-5-3-5,5-3-3-10). The results demonstrated that the dose–response curve was linear in the dose range of 0 to 6 Gy, and the linearity of the two NIPAM gel formulations at the depth of the dose point (2 cm) was 0.98–0.99.

Previous studies have shown that sensitivity is the slope of the dose–response curve [20]. In the current study, Fig 4 shows that the sensitivity of the 5-5-3-5 formulation was 0.0022 cm^−1^ Gy^−1^, while that of the 5-3-3-10 formulation was 0.001 cm -1 Gy -1 for Proton therapy.

The present study found that the sensitivity of the gel dosimeter was relatively small compared to that in previous photon therapy studies with the same recipe of NIPAM gel.

### Percentage depth dose

**Fig 5:**
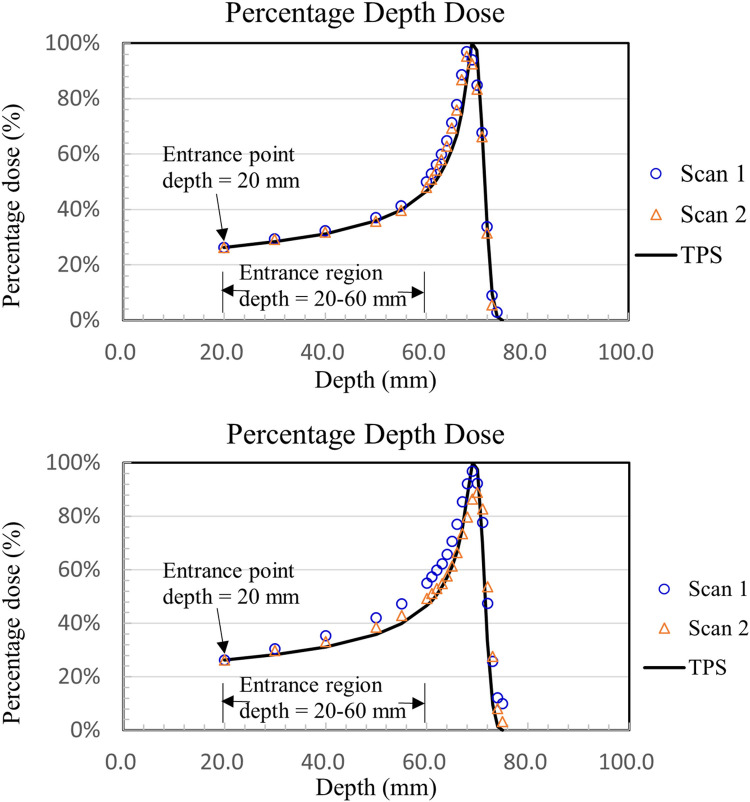
Percentage depth dose of low-dose irradiation (1–3 Gy), (a) recipe 5-3-3-10, low sensitivity formulation; (b) recipe 5-5-3-5, high sensitivity formulation.

**Fig 6:**
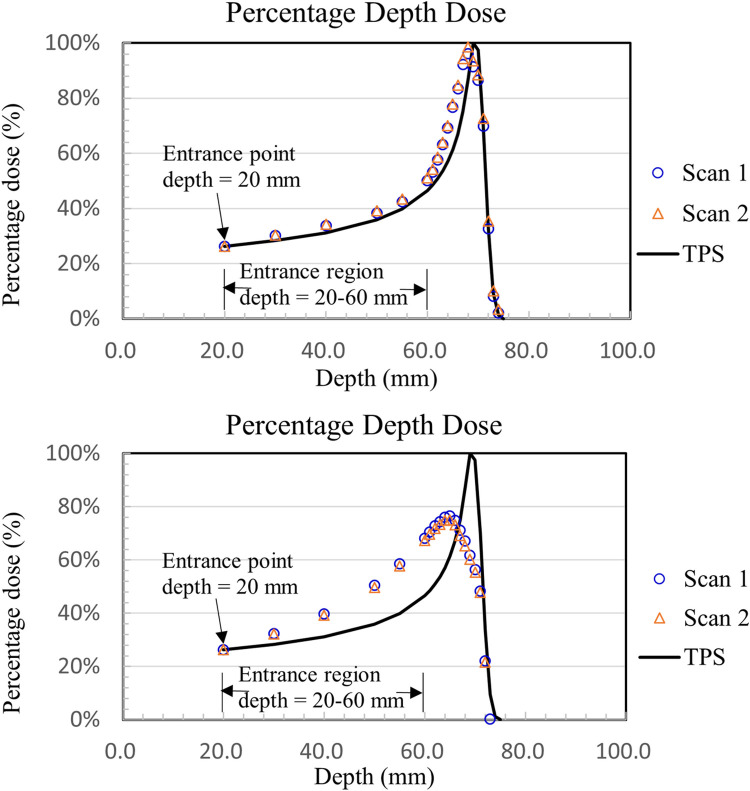
Percentage depth dose of high-dose irradiation (4 to 6 Gy), (a) recipe 5-3-3-10, low sensitivity formulation; (b) recipe 5-5-3-5, high sensitivity formulation.

The comparisons between the calculated TPS result and gel measurement and the percent depth dose curve after normalizing the data to a depth of 2 cm are shown in Figs 5 and 6.

Fig 5 shows that the NIPAM gels of the two formulations were irradiated with a low dose (1–3 Gy), and the Bragg peak in the percentage depth dose curve was underestimated to be approximately 4% and 7% compared to the absorbed dose value calculated using TPS.

In the beam entrance region before the Bragg peak (20–60 mm depth), the differences between the measured NIPAM value and the depth dose curve calculated using TPS were 7% and 5%.

Fig 6 shows the NIPAM gels of the two formulations after high-dose (4 to 6 Gy) irradiation compared to the absorbed dose of TPS. The results showed that the absorbed doses of the NIPAM dosimeter were underestimated to be approximately 4% and 25%. The differences between the measured NIPAM value and the depth dose curve calculated using TPS were 8% and 13% in the beam entrance region (2–6 cm depth).

The study found that the two formulations of NIPAM gel dosimeters after exposure to proton beams, the highly sensitive formulations were underestimated compared to TPS at high-dose irradiation (4 to 6 Gy), but at low doses (1–3 Gy) is similar to the less sensitive formula. The formula with low sensitivity under 1–6 Gy is consistent with the TPS proton depth dose.

The Bragg peak underestimate was similar to the results observed in previous research on the application of normoxic polymer gel dosimeters to proton therapy due to the LET and quenching effects [12, 13, 15, 16, 21, 22]. Gustavsson et al. [13] mentioned that the yield of free radicals decreases with increasing LET, leading to a decrease in the polymerization reaction and an underestimation of the gel dose.

### Temporal stability

**Fig 7:**
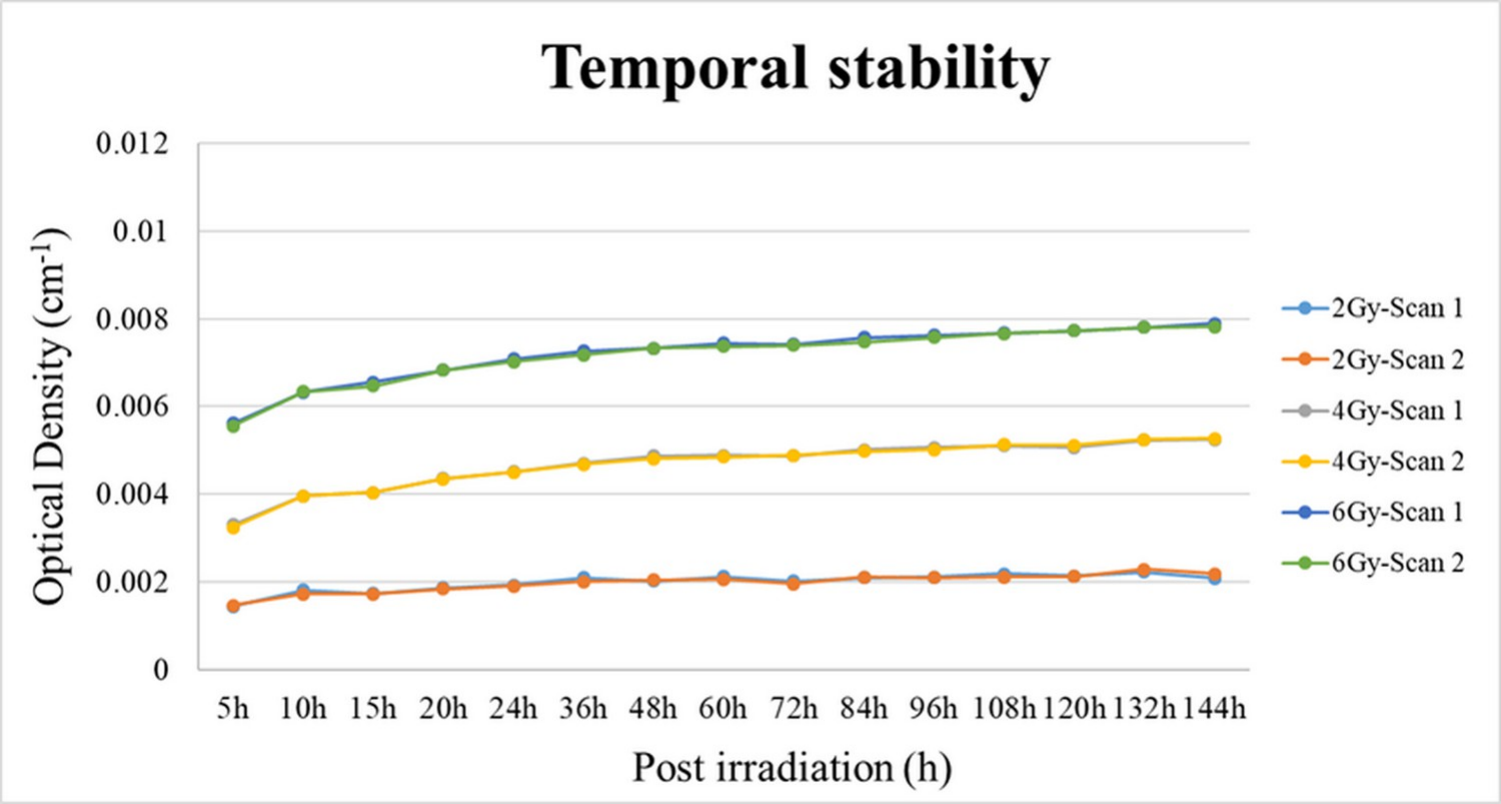
Optical density values at different time points after irradiation of the NIPAM polymer gel.

The polymer gel dosimeter is one of the chemical dosimeters, and the polymerization reaction changes with time [23, 24]. Therefore, the present study explored how to obtain a more accurate dose when the gel was used for proton therapy. OD values at 5, 10, 15, 20, 24, 48, 72, 96, 108, 120, 132, and 144 hours were measured after NIPAM gel irradiation, as shown in Fig 7.

In this Fig 7, the chemical polymerization of the NIPAM gel dosimeter was sustained until 48 h. However, previous studies have reported that chemical polymerization is stable for 24 h.

### Dose-rate dependence

The dose-rate dependence of the gel was investigated by exposing the gel to three different target doses of 2, 4, and 6 Gy and dose rates of 20, 40, and 80 MU/S for a 100 Mev proton beam.

**Fig 8:**
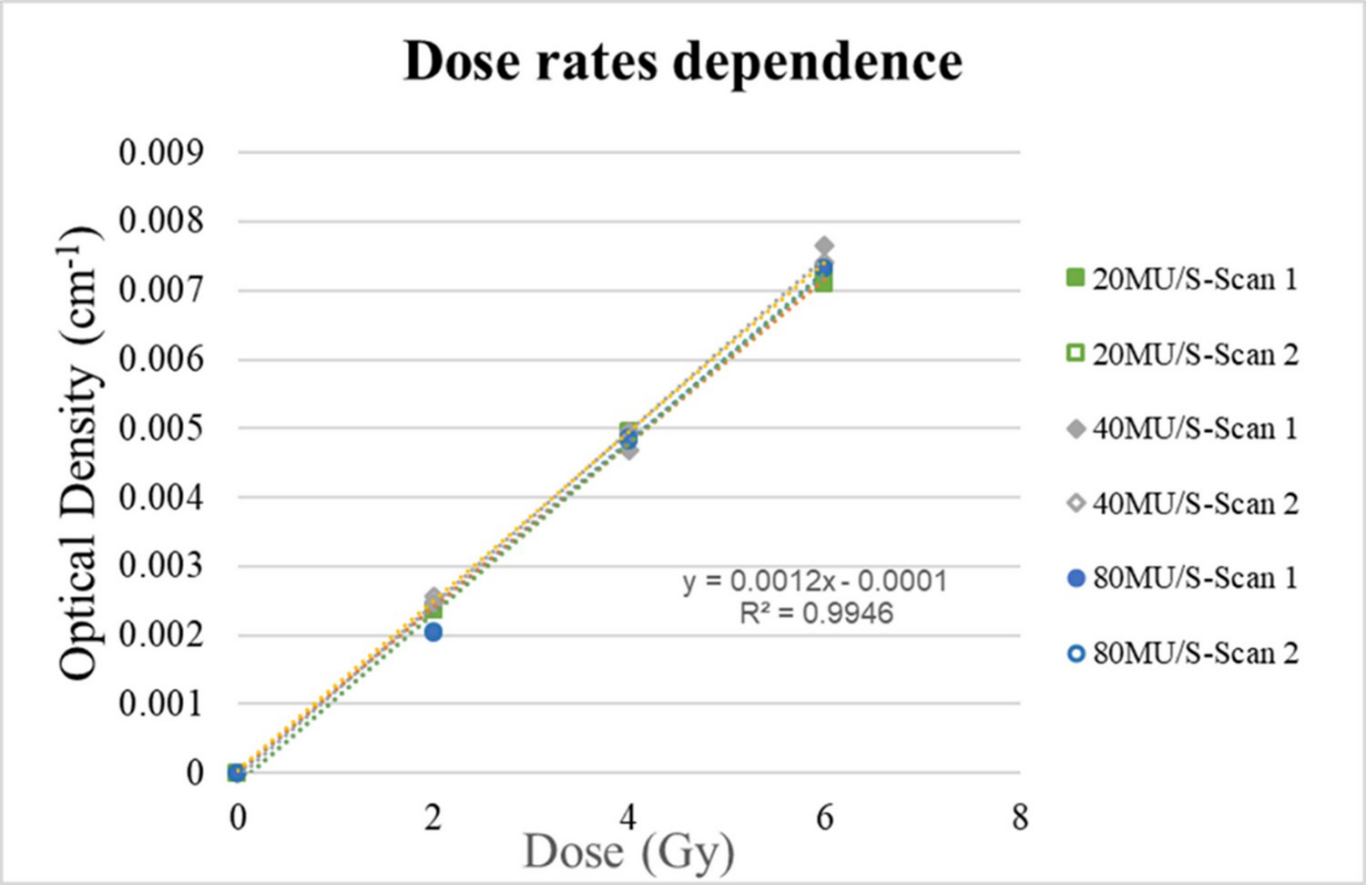
Dose-rate dependence of NIPAM polymer gel dosimeters at different dose.

Fig 8 illustrates a graph of the relationship between the OD value and the different dose rates at a fixed target dose and a gel depth of 20 mm. There were nine batches of gel dosimeters irradiated with three dose rates of irradiation, that is, 20 MU/s, 40 MU/s, and 80 MU/s and three target doses, that is, 2 Gy 4 Gy, and 6 Gy, as listed in Table 2. Each gel dosimeter was irradiated with one dose rate and one target dose, but was scanned twice. This result indicated that the NIPAM polymer gel dosimeter had the smallest dose-rate dependence, and the R^2^ value of this linearity was 0.99.

### Energy dependence

In the energy-dependent experiment, a unirradiated gel was used as 0 Gy, and three doses of 1, 2, and 3 Gy were fixed. Each dose was irradiated with three different energies of 80, 100, and 120 MeV.

**Fig 9:**
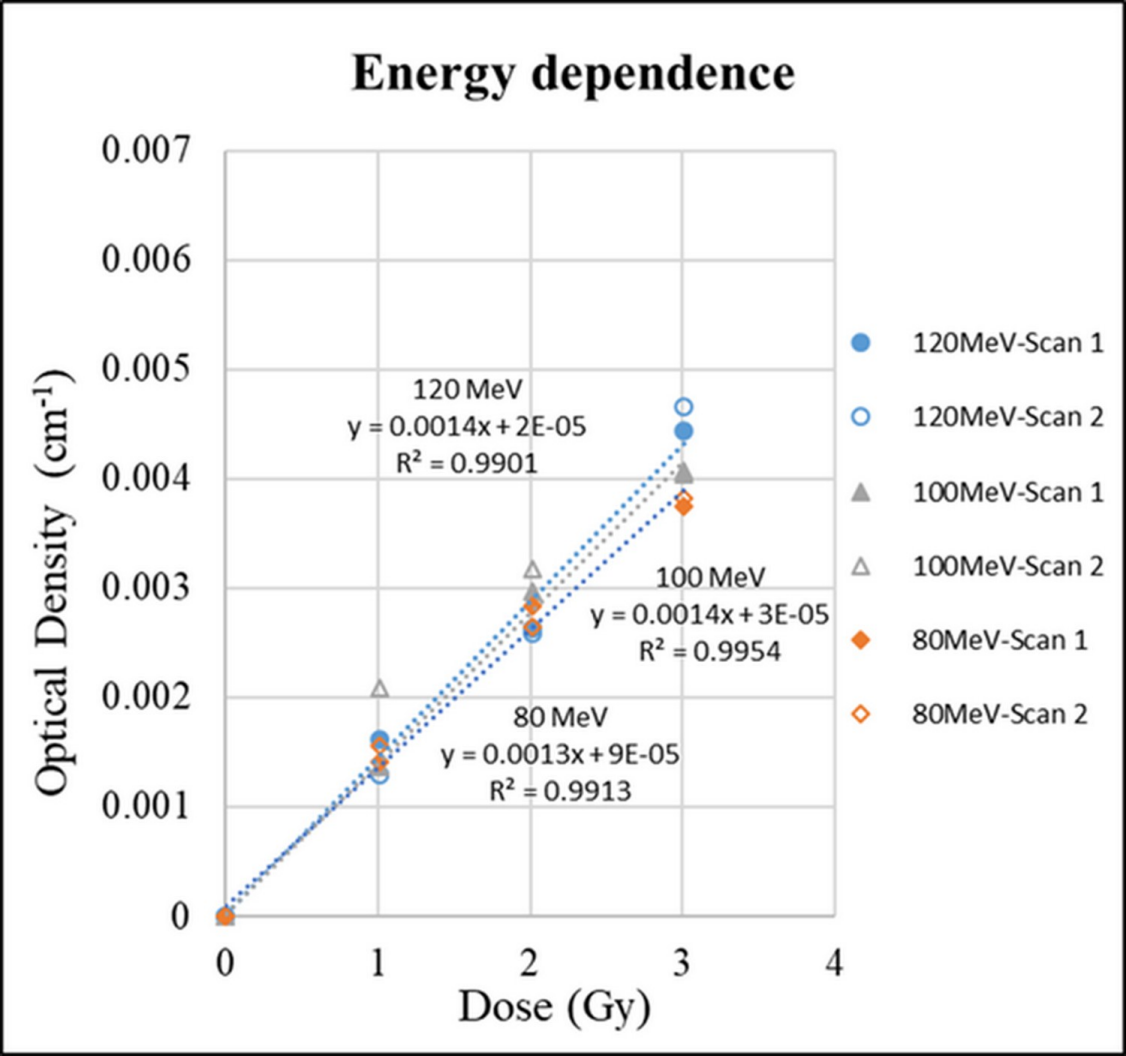
Energy dependence of NIPAM polymer gel dosimeters at different energies.

Fig 9 shows the linear relationship between the OD value and the different energies at a fixed target dose and a gel depth of 2 cm. The results showed that the dose sensitivities (linear slope) of 80, 100, and 120 MEv were 0.0013, 0.0014, and 0.0014 Gy^−1^min^−1^. When comparing the dose sensitivities of 80 and 100 MeV with 120 MeV, the maximum difference was only 7%. Therefore, the NIPAM gel dosimeter had no energy dependence problem.

## Discussion

The NIPAM gel dosimeter is known as one type of chemical dosimeter. To ensure its use in the clinic, the gel dosimeter must possess suitable sensitivity and high linearity. Various NIPAM gel formulations have been proposed and validated by various linear accelerators [4–11]. However, the sensitivity and linearity may vary due to the chemical chain reaction of the polymer gel when irradiated by different radiation beams, such as proton beams and linear accelerators. Therefore, we had to prepare a suitable gel dosimeter that demonstrated proper sensitivity and linearity when used for different radiation types.

In the current study, the NIPAM gel dosimeter was applied to proton therapy. The results showed that the sensitivity and linearity of the proposed NIPAM gel dosimeter were suitable for proton therapy. Additionally, the proposed NIPAM gel dosimeter is energy independent and dose rate independent, both of which are crucial factors for a reliable dosimeter. This ensures that a chemical dosimeter can exhibit the same response when subjected to different treatment planning system (TPS) radiations.

Another highly important factor for a good dosimeter is temporal stability, which guarantees that a chemical dosimeter can retain the absorbed dose for a certain period. The results indicated that the NIPAM gel dosimeter remained stable from 24 h to 144 h.

One advantage of 3D gel dosimeters for proton therapy is their capability to indicate the location of the Bragg peak, as shown in Figs 5 and 6. The percentage depth dose results demonstrated a highly accurate dose response of the NIPAM gel dosimeter when irradiated with 1–3 Gy for both gel recipes, namely 5-3-3-10 (low sensitivity formulation) and 5-5-3-5 (high sensitivity formulation). The differences between the measured values of NIPAM and the treatment planning system (TPS) were 7% (Fig 5(A)) and 5% (Fig 5(B)), respectively.

Furthermore, when the NIPAM gel dosimeter was irradiated with 4–6 Gy, the gel recipe 5-3-3-10 (low sensitivity formulation) still exhibited a highly accurate dose response. The differences between the measured NIPAM value and TPS were 8% (Fig 6(A)). However, the NIPAM value of the 5-5-3-5 gel recipe (high sensitivity formulation) showed a poor dose response, with differences up to 13% (Fig 6(B)). The dose value measured by the NIPAM gel dosimeter was underestimated by approximately 25% near the Bragg peak. This could be attributed to the quenching effect of linear energy transfer (LET), which decreases the formation of free radicals and leads to a decrease in the polymerization reaction [13].

## Conclusions

The dose-response curve at the depth of 2 cm of the high- and low-sensitivity preparation gel shows that the curve between OD and absorbed dose is linear in the dose range of 0–6 Gy.

In addition, in terms of curve fitting percentage, there is no significant difference between the entry dose (20–60 mm) and clinical data for either high-sensitivity or low-sensitivity formulations. However, the high-sensitivity preparation group reached saturation at the Bragg peak, where the dose was higher, causing a serious underestimation.

Therefore, proton therapy is not suitable for selecting gel formulations with high sensitivity when achieving more accurate dose distribution and obtaining the best scan results.

The study also explored polymer gels at different dose rates and energies. The results show that the slopes of the dose-response curve are consistent, which confirms that the NIPAM dosimeter has no energy and dose rate dependence problems under proton beam irradiation.

The results of the study show that it is feasible to use the NIPAM dosimeter for proton therapy. Future research may focus on the polymerization mechanism after chemical polymer irradiation and dose verification experiments, as clinical needs increase.

## Supporting information

S1 File Dose–response curves. (XLSX)

S2 File Percentage depth dose of low-dose irradiation. (XLSX)

S3 File Percentage depth dose of high-dose irradiation. (XLSX)

S4 File Optical density values. (XLSX)

S5 File Dose-rate dependence. (XLSX)

S6 File Energy dependence. (XLSX)
